# Geographic and ecologic heterogeneity in elimination thresholds for the major vector-borne helminthic disease, lymphatic filariasis

**DOI:** 10.1186/1741-7007-8-22

**Published:** 2010-03-17

**Authors:** Manoj Gambhir, Moses Bockarie, Daniel Tisch, James Kazura, Justin Remais, Robert Spear, Edwin Michael

**Affiliations:** 1 Department of Infectious Disease Epidemiology, Imperial College London, UK; 2 Liverpool School of Tropical Medicine and Hygiene, University of Liverpool, UK; 3 Center for Global Health and Diseases, Case Western Reserve University, OH, USA; 4 Rollins School of Public Health, Emory University, GA, USA; 5 School of Public Health, University of California, Berkeley, CA, USA

## Abstract

**Background:**

Large-scale intervention programmes to control or eliminate several infectious diseases are currently underway worldwide. However, a major unresolved question remains: what are reasonable stopping points for these programmes? Recent theoretical work has highlighted how the ecological complexity and heterogeneity inherent in the transmission dynamics of macroparasites can result in elimination thresholds that vary between local communities. Here, we examine the empirical evidence for this hypothesis and its implications for the global elimination of the major macroparasitic disease, lymphatic filariasis, by applying a novel Bayesian computer simulation procedure to fit a dynamic model of the transmission of this parasitic disease to field data from nine villages with different ecological and geographical characteristics. Baseline lymphatic filariasis microfilarial age-prevalence data from three geographically distinct endemic regions, across which the major vector populations implicated in parasite transmission also differed, were used to fit and calibrate the relevant vector-specific filariasis transmission models. Ensembles of parasite elimination thresholds, generated using the Bayesian fitting procedure, were then examined in order to evaluate site-specific heterogeneity in the values of these thresholds and investigate the ecological factors that may underlie such variability

**Results:**

We show that parameters of density-dependent functions relating to immunity, parasite establishment, as well as parasite aggregation, varied significantly between the nine different settings, contributing to locally varying filarial elimination thresholds. Parasite elimination thresholds predicted for the settings in which the mosquito vector is anopheline were, however, found to be higher than those in which the mosquito is culicine, substantiating our previous theoretical findings. The results also indicate that the probability that the parasite will be eliminated following six rounds of Mass Drug Administration with diethylcarbamazine and albendazole decreases markedly but non-linearly as the annual biting rate and parasite reproduction number increases.

**Conclusions:**

This paper shows that specific ecological conditions in a community can lead to significant local differences in population dynamics and, consequently, elimination threshold estimates for lymphatic filariasis. These findings, and the difficulty of measuring the key local parameters (infection aggregation and acquired immunity) governing differences in transmission thresholds between communities, mean that it is necessary for us to rethink the utility of the current anticipatory approaches for achieving the elimination of filariasis both locally and globally.

## Background

Large-scale intervention programmes to control or eliminate a group of tropical infectious diseases are currently underway in many parts of the world [1,2]. These neglected tropical disease (NTD) control programmes are primarily based on the administration of highly effective drugs to entire afflicted populations, although additional measures, such as vector control and sanitation, often accompany the drug distribution [3]. These diseases have been prevalent in tropical and sub-tropical regions for millennia [4] and have been shown to be very difficult to bring under control so that, following the termination of previous control efforts, infection and disease often reemerge in endemic populations [5,6]. Recent theoretical work has highlighted how the difficulty in achieving the elimination of infection may be related to the ecological complexity and heterogeneity inherent in the transmission dynamics of the parasites causing these NTDs [7,8].

Two important threshold values that govern the switching of dynamic vector-borne helminth systems from one stable state to another [8,9], either settling at a stable endemic or extinction steady state, are the threshold biting rate (TBR; the vector biting rate below which infection cannot be sustained in the population) and the worm breakpoint (the host parasite prevalence below which local extinction occurs) [8,10]. Depressing infection or biting rate levels below these thresholds (by promoting the 'good' transition from stable infection to parasite elimination [9]) is the objective of any elimination programme. Mathematical models, based on the dynamic mechanisms by which vector-borne helminth infection occurs, provide an important tool for the calculation of the TBR and parasite breakpoint values [7,8,11]. However, the likelihood that local parasite transmission dynamics will differ from one community to another means that reliably estimating the values of these thresholds will require the efficient fitting of models to site-specific infection data. Such data-driven model-based estimation is also necessitated by the often large number of uncertainties associated with the model structure, parameterization (especially when such models are characterized by a relatively large number of parameters, as is typical with dynamic parasite transmission models) and prediction [12-16]. For these reasons, the widespread use of process-based models for guiding parasite control based on theoretical predictions has so far been limited.

Also, fitting complex ecological models to data is not a trivial task [16], especially when there is uncertainty and a lack of detail in the site-specific infection data available for reliable model parameter estimation. Thus, in recent years an increasing focus in work relating to dynamic process-based models for practical applications has been on the development and application of fitting procedures that can allow the use of information from available data to refine and update initially assigned model parameter values [12-15,17,18].

Our aims here are threefold. First, we fit a mathematical model of lymphatic filariasis (LF) transmission- against which a global elimination programme is currently underway- to community age-prevalence data from three geographical regions, where two different mosquito species transmit the parasite, to test the hypothesis that elimination thresholds for this major vector-borne disease vary significantly between communities [8]. Second, we use site-specific data, and a recent approach based on fitting dynamic parasite transmission models to data via computer simulation techniques [15], to update our current knowledge of parameter values (and, hence, enhance our knowledge of key parasite transmission processes) and quantify the extant uncertainty around elimination breakpoint values. Finally, we analyse model parameter values estimated from each study area in order to investigate the factors that underlie the observed between-community variation in these elimination thresholds. We end by showing the importance of the present results for the current World Health Organization (WHO) strategy for eliminating LF based on annual mass chemotherapy, by quantifying, given the estimated breakpoint values for a community, the probability of achieving infection elimination locally by deploying the currently recommended global WHO mass treatment regimen.

## Results

### Data and fitted mf age-prevalence curves for each study community

**Figure 1:**
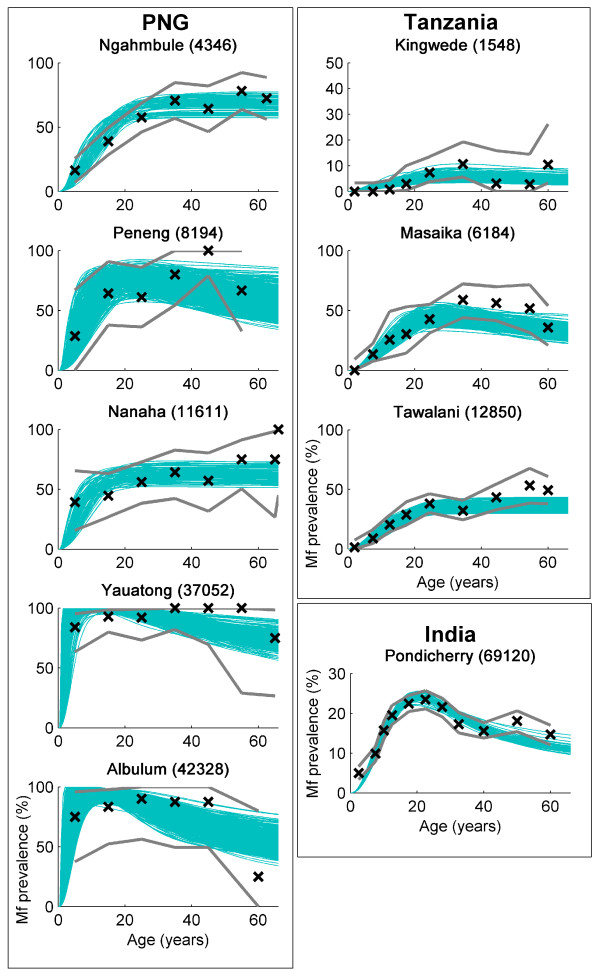
**Observed and fitted microfilarial age-prevalence curves for each endemic setting**. The 500 curves (blue solid lines) generated by importance resampling of the input parameter sets according to their likelihood are displayed against observed data (black crosses with solid black lines showing upper and lower 95% confidence intervals of the data) for each of the communities investigated in this study. Five hundred unique curves may not be seen in each case since those with the highest likelihood will be plotted several times. Each of the curves represents an equilibrium solution to the model described in the text, given the actual annual biting rate (values in parentheses) obtained in each community. The appropriate vector uptake function (corresponding to the *Anopheles *or *Culex *species) was used in each case. Country and village names are given above each plot.

The 500 age-dependent equilibrium curves obtained by resampling the original parameter sets using the sampling importance resampling (SIR) algorithm (see Methods) are plotted against observed microfilarial (mf) data in Figure 1 for each of the data sets studied here. The results show that, over the range of annual biting rates (ABR) found between the study sites and for the two different mosquito vectors, the present models are capable of reproducing mf prevalence curves consistent with observed data. Each of the curves is generated by a different model parameter set and the range of curves produced represents the residual uncertainty remaining in the parameters following the Bayesian updating procedure. Note that 500 distinct curves are unlikely to be plotted in each case as those curves with the highest likelihoods will be plotted multiple times.

### Parameter values

**Figure 2:**
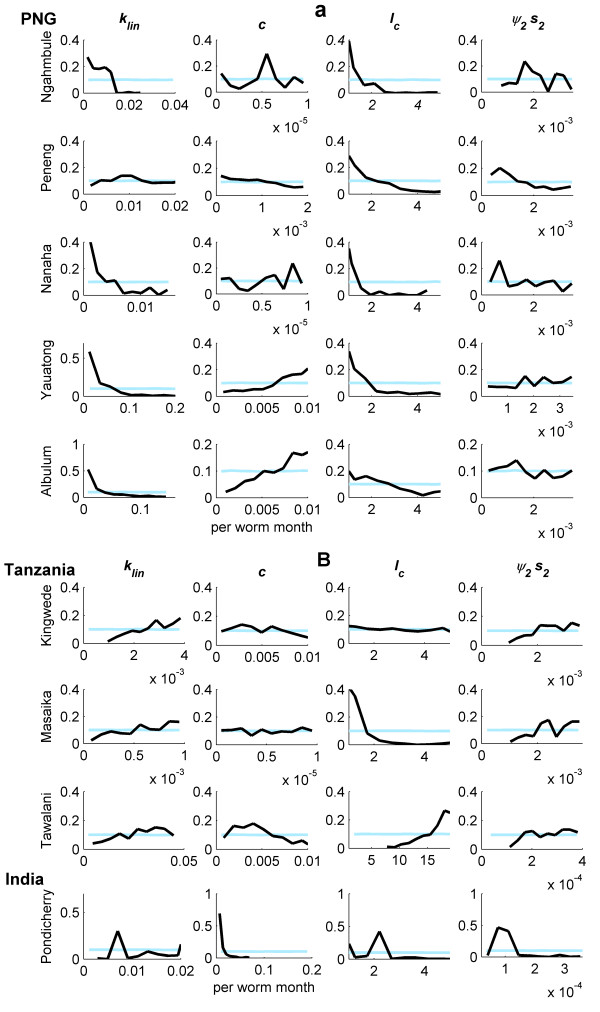
**Prior and posterior model parameter distributions for the data from each community**. A comparison of the prior (light blue lines) and posterior (black lines) parameter relative frequency distributions obtained from model fits to age-mf prevalence data in each of the study villages (the village name is given on the far left of each row of four graphs). The parameters illustrated - *k*_*lin*_: linear component of the mf aggregation parameter; *c*: strength of immunity to larval establishment; *I*_*C*_: strength of immunosuppression; *ψ*_2 _*s*_2_: referred to as the establishment rate, all of which are dimensionless except for *c *(see Additional File 1, Tables 1 and 2) - are those that showed a strong difference between the initially assigned uniform 'flat' priors and the obtained posteriors within a data set. Graph (a) shows results for Papua New Guinea villages; and (b): Tanzania and India villages.

As noted above, an important application of the Bayesian model fitting procedure we use is to produce better estimates of the parameters underlying LF transmission. Figure 2 shows comparisons of the initially assigned flat, or uninformative, prior and the obtained posterior distributions for four of the model parameters following the application of the Bayesian updating procedure to the data from each of the study villages. As their posterior distributions differed strongly from their priors (posterior distributions becoming distinctly non-flat), these parameters were illustrated in Figure 2 to provide a clear depiction of how values of a parasite transmission parameter can be effectively refined or updated by applying the present Bayesian melding (BM) procedure to field infection data. More formal comparisons between the prior and posterior distributions of each model parameter for each of the study data sets were conducted using the univariate Kolmogorov-Smirnov statistic and are given in Additional File 1 (Table 3) online. An important finding depicted by these results is that increasing changes in the posterior distributions, relative to initially assigned uninformative priors, will occur as the variability in observed data decreases. Thus, all of the posterior distributions were significantly different from the assigned uniform priors for the Pondicherry data set, which exhibited the lowest variability in mf prevalence with age (as a result of its bigger sample size), with this comparative difference declining markedly in the case of the more variable Papua New Guinea (PNG) data sets.

### Biting thresholds and worm breakpoints

**Figure 3:**
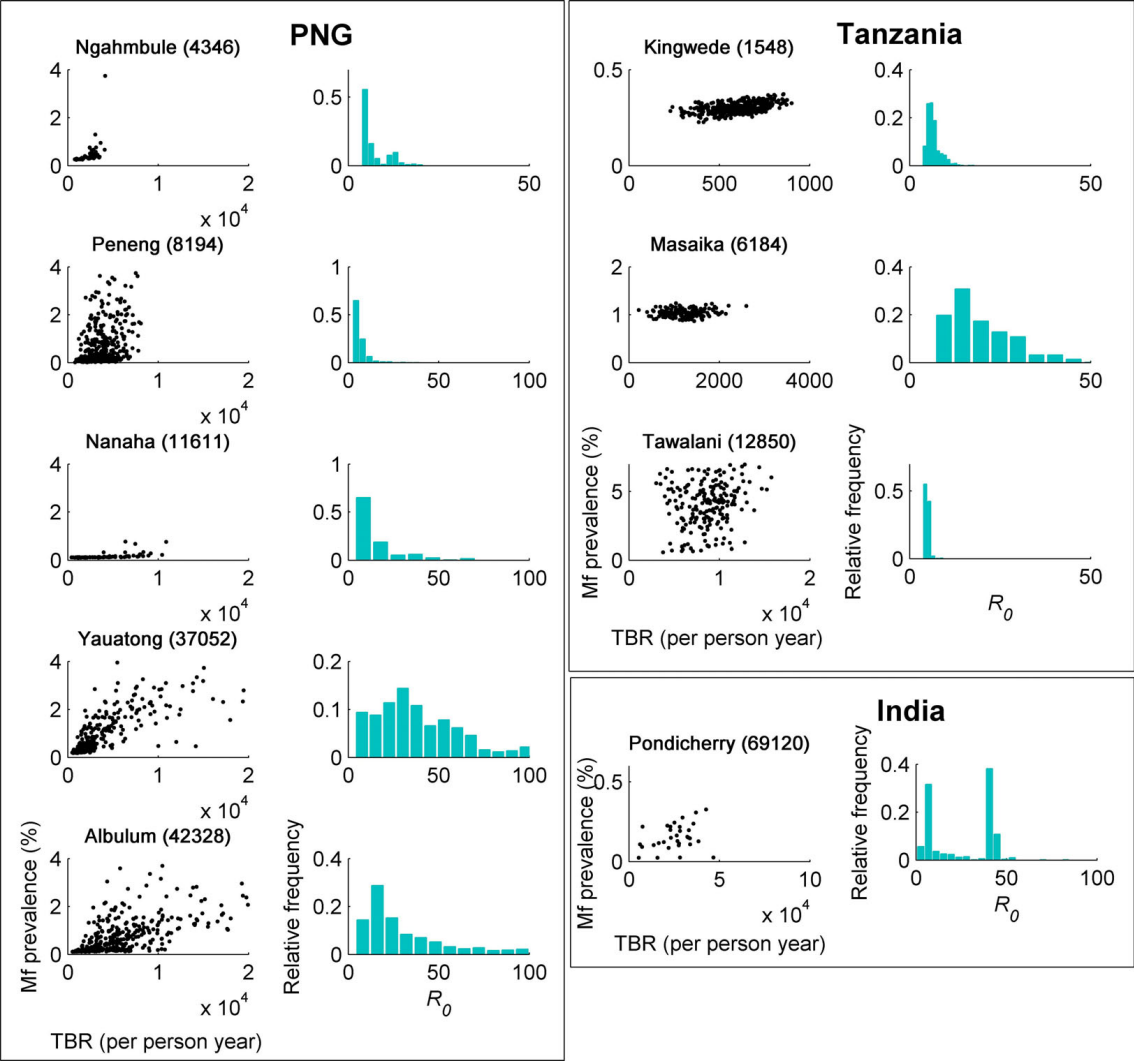
**Breakpoint and reproduction number estimates**. Breakpoints versus threshold biting rates (TBRs; black scatter plots) and the estimated *R*_0 _(histograms) for the best-fitting parameter sets obtained from each of the data sets investigated in this study. Country and village names are given above each plot, along with annual biting rate values in parentheses.

Figure 3 shows ensembles of the maximum values of the breakpoints and their corresponding TBRs obtained as a result of model fits to each of the data sets. In addition, a histogram of the corresponding *R*_0 _values for each study community (see Methods and Additional File 1) is also shown. For anopheline-transmitted filariasis where, due to a positive density dependence, the basic reproduction number is hard to define (see Additional File 1) we follow Regoes *et al*. [19] in calculating a threshold reproduction number pertaining to the steepest part of the mf-L3 uptake curve for this mosquito, which can be defined to be equivalent to the reproduction number in culicine systems. The results depicted in Figure 3 - for the TBRs, mf prevalence breakpoints and *R*_0_- show not only that a wide distribution of values is consistent with the data in each study community when the model is fitted to mf age prevalence but also that they can vary significantly between the study communities [kruskal-wallis tests of differences in estimated values of each variable indicating highly significant study effects in each case (*P *< 0.0001 in each case)].

**Figure 4:**
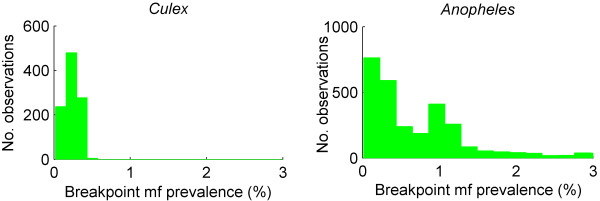
**Breakpoint comparisons across the two mosquito species**. Histograms of the distribution of breakpoints, calculated for the accepted parameter sets and aggregated for data sets in which the vector is culicine or anopheline. The median breakpoint value for the culicine model fits is 0.23% microfilaria (mf) with 95% of values lying above 0.09% mf, whereas for anopheline models the median value is 0.75% with 95% of values lying above 0.12%.

Figure 4 contains a pair of histograms of the mf prevalence breakpoints, aggregated over the study data according to whether the vector is culicine or anopheline. The histograms show that the anopheline breakpoints are much more widely distributed than those for culicine mosquitoes. The medians of the two prevalence distributions were 0.76% for the combined anopheline model-generated results and 0.23% for the culicine models, which supports previous theoretical results that worm breakpoints will be markedly higher in the case of anopheline compared to culicine filariasis [8]. Interestingly, the present data-driven analysis indicated that, whereas the observed difference in the estimated mf breakpoint values was statistically significant between the two vector species (kruskal wallis χ^2 ^= 742.2105, df = 1, *P *< 0.0001), the corresponding estimates for TBR and *R*_0 _were not.

**Figure 5:**
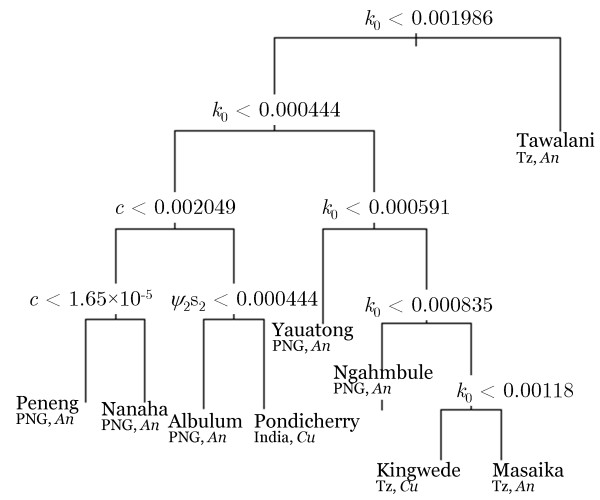
**Classification tree model showing the model parameters that differed significantly between the various study sites**. The results show that differences in the infection aggregation parameter (*k*_0_), signifying how over dispersed infection among individuals in each community was (lower value higher the overdispersion), the acquired immunity parameter (*c*) and the establishment rate (*ψ*_2_*s*_2_), primarily underlay the variations observed in the parameter vector estimates obtained between the study communities investigated in this study. The cross-validated error rate of the displayed model (with eight splits) was low at approximately 1%. The classification tree was fit using the *rpart *package in R.

We investigated which of the fitted model parameters differed significantly between the study communities and, therefore, may underlie the between-study variations observed for the estimated breakpoints and *R*_0 _values (shown in Figure 3) via classification tree analysis. Figure 5 displays the final fitted tree and indicates that, of the various model parameters (see Additional File 1, Tables 1 and 2), variations in the fitted community infection aggregation parameter (*k*_0_), acquired immunity (*c*) and parasite establishment rate in the human host (*ψ*_2_*s*_2_) may primarily contribute to the differences observed in the infection dynamics and, hence, the breakpoint and *R*_0 _values estimated between the different study communities investigated here. The results also suggest that differences in the community infection aggregation index, by contributing to many of the earlier splits in the tree, constitute the most important factor that may influence the observed differences in parameter vectors or infection dynamics between the study villages followed, less significantly, by the immunity and parasite establishment parameters. Note also that, while the aggregation and immunity variables may constitute site-specific parameters that could be expected to vary between communities [8,20,21], the establishment rate parameter, by contrast, is an intrinsic biological parameter, which may differentially influence LF transmission between communities - possibly as a result of interactions with site-specific parameters, such as immunity [20,21].

### Impact of locally applicable breakpoints on annual repeated mass drug administration (MDA) programmes

**Figure 6:**
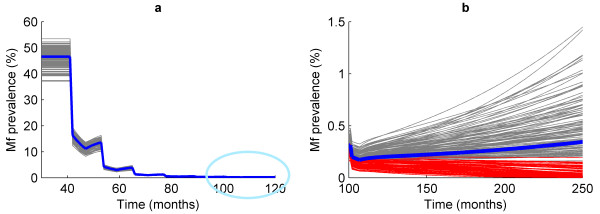
**Extinction and re-emergence of infection following six rounds of mass drug administration**. The effect of six annual mass drug administration (MDA) rounds on 500 accepted or passing parameter sets of the model fitted to baseline age-mf prevalence data of the Papua New Guinea village, Ngahmbule. The figure on the left (a) shows that the response to six MDAs is very similar in each realization, with the prevalence dropping to very low levels after the final treatment round. The figure on the right (b) zooms in on the region encircled by the light blue line; it shows that, following six treatments, only approximately half of the 500 trajectories are sufficiently reduced in mf prevalence to have dropped below the breakpoint and for the parasite to go to extinction.

The current WHO-recommended strategy for filariasis control is based on the expectation that six annual doses of drug treatment could eliminate LF from a community. We simulated the impact of a combined annual mass diethylcarbamazine/albendazole (DEC/ALB) regimen applied at 80% coverage (using efficacy estimates given by Michael *et al*. [22]). The analysis was carried out by subjecting each of the resampled 500 parameter sets from each village to the recommended regime of six annual DEC/ALB treatments, after which we determined the proportion of the 500 model simulations that crossed their mf prevalence breakpoints. Figure 6 plots the trajectories followed by each model run for the PNG village of Ngahmbule and shows that, although the overall mean mf prevalence calculated over all of the 500 model realizations may fall to very low levels following the six rounds of treatment, approximately half of the 500 model runs resulted in a decrease in the parasite intensity and prevalence to a level whereby extinction occurs without further treatment.

**Figure 7:**
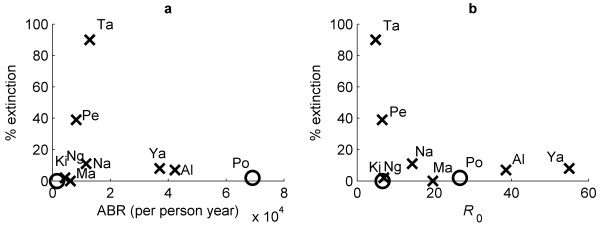
**Parasite extinction rates following six mass drug administration (MDA) rounds**. Both graphs show the outcome of six MDA rounds at 80% coverage on the 500 passing parameter sets for each study community. Graph (a) shows the relationship between the proportion of the 500 accepted models that go to extinction - that is, the microfilaria prevalence drops below the breakpoint estimated for each individual accepted model, and the annual biting rate of the corresponding study community, while the graph (b) plots the same proportions of the models that go to extinction but in relation to the mean value of *R*_0 _for each community. Black crosses correspond to anopheline models and circles are culicine.

Figure 7 shows the percentage of the 500 best-fitting model runs from each of the study communities that went to extinction in relation to both the observed study-level ABR (Figure 7a) and estimated mean *R*_0 _values (Figure 7b). Both graphs show that the probability of LF extinction by annual MDA for a fixed treatment duration (the WHO-recommended 6 years) varied markedly between the study communities, although in a manner wherein this probability declined strongly, and non-linearly, with increasing community ABR or with the increasing internally-derived related variable, *R*_0_, of the parasite. The non-linearity shown for these relationships in Figure 7, however, indicates that above a particular ABR or *R*_0 _threshold, LF extinction rates among the study communities were similar, despite a wide range of initial ABR or *R*_0 _values obtaining in the higher endemic communities. This association is slightly more variable with observed community ABR values compared to the model estimated *R*_0 _values for each study community.

## Discussion

The major result of this study of immediate import to LF elimination is our finding of the occurrence of significant differences in the population dynamics and the resulting transmission breakpoint estimates between the nine endemic villages investigated. Although differences in the transmission dynamics of this parasitic disease have been investigated before, they have primarily focused on uncovering the impact of *a priori *proposed drivers of such differences, such as community vector biting rates and acquired immunity [20,21,23,24]. However, this study is the first to use empirical data to disclose the key transmission parameters that underlie observed site-specific differences in filarial transmission dynamics and the resulting endpoints for terminating parasite transmission. The results highlight two important conclusions on this point. First, they support previous results, from sensitivity analyses of our model, that transmission breakpoints in each of the two major filarial infection systems are likely to be highly sensitive to variations in site-specific ecological factors underlying infection dynamics. Second, they confirm that such factors are primarily related to the degree of infection aggregation, as well as the magnitude of acquired immunity occurring within endemic communities [8], although differences in the parasite establishment rate- a more intrinsic biological parameter, the values of which are likely to depend on the strength of immunity operating in a community [20] - may also, to a lesser degree, govern between-community differences in the values of such breakpoints.

The Monte Carlo-based sampling technique used for model fitting has also allowed the first estimates of the extant uncertainty in breakpoint values for eliminating LF. Thus, we found, for example, that worm breakpoints, when aggregated over the models for each vector, resulted in a higher spread of values for the anopheline than the culicine models (the median mf prevalence breakpoint value was 0.76% for anopheline models as opposed to 0.23% for culicine). Although this vector-specific discrepancy in the median value for this breakpoint has previously been suggested [8,11] (and is due to the presence of two facilitation density dependences (mosquito uptake and worm mating probability) for the anopheline against only one (mating probability) for the culicine-mediated filarial infection dynamics), the characterization of variability in the breakpoint values, even within a local setting, is a new outcome of this study. While the uncertainty estimates for both breakpoints and for *R*_0 _obtained in this study primarily reflect epistemic uncertainty regarding parameter values and their distributional patterns (which we expect to refine by model updating with data), the possible existence of a range or cloud of extinction breakpoints within a setting, nonetheless, supports the notion that stochastic variability in infection parameters will, in reality, give rise to a distribution rather than a simple point estimate for these variables in natural communities [10,25]. Nevertheless, the data-driven finding here substantiates the theoretical conjecture [7,8] that it may be easier, if all other factors are held constant, to eliminate anopheline rather than culicine filariasis in the field. Future work should include further data sets for both culicine and anopheline-mediated filariasis in order to increase the statistical validity of these findings.

Our simulations of the impact of the WHO-recommended drug administration strategy (six annual mass treatments with either DEC/ALB or invermectin/ALB and a population coverage of 80%) demonstrate the likely failure of a fixed global strategy that ignores local extinction dynamics. Not only may such a strategy produce a great deal of site-to-site variability in the prospects of achieving filariasis elimination but a consideration of the drivers of transmission, such as community ABR and *R*_0 _values, may also have limited usefulness in predicting the likely success of time-bound intervention strategies for accomplishing parasite elimination, especially in those areas where the values of both these variables are high. The importance of this result for filariasis elimination programmes is clear: because the complex dynamics governing parasite transmission may cause filarial transmission breakpoints to vary between communities, any effort which aims to achieve the elimination of this disease must be based on estimates of local thresholds [8,15,26].

The results of our parameter estimation have demonstrated that, although a greater knowledge of the natural variability occurring in key filariasis transmission parameters can clearly be gained using the BM model fitting approach, successful parameter updating is critically related to the quality of the available data. Thus, the most informative data are those that show low levels of variability, suggesting that in order to be useful, parameter estimation requires that either good quality data are collected and subjected to analysis or else a hierarchical multi-level framework should be developed that allows the combining of data from different communities with as similar transmission characteristics as possible. The Bayesian approach employed here will also allow for the future inclusion of further information or data, such as treatment follow-up data from local sites, which may be used sequentially to refine the model-fitting process and, hence, update parameter estimates [27]. Such updating of the present models with more site-specific and follow-up data may also eventually enable us to determine which components, or even model structure, is necessary to obtain the most parsimonious description of the host-parasite system in different endemic localities [28].

There are further limitations to our modelling approach which need to be borne in mind when interpreting the present results. The most important of these is that, although our deterministic modelling framework has yielded important insights into the extinction dynamics of LF as a result of mass drug interventions, stochastic analogues of our models would clearly enable the investigation of a greater number of sources of extinction, including the role of pure demographic effects and the impact of external drivers of population dynamics such as varying environmental or climate variables. In addition, future work must not only provide a better understanding of the forms and parameter values of the density-dependent processes that need to be included in the model to explain data in different communities, but must also show how these functions may, in turn, interact with different interventions in order to govern the specificity of the parasite population response to control. The use of longitudinal follow-up data in conjunction with model updating procedures, such as the Bayesian estimation procedure described here, will allow an analytical framework to achieve this objective.

Despite these caveats, the present findings point to important implications for the design of filariasis elimination programmes. First, the difficulty of measuring the key local parameters (for example, infection aggregation, acquired immunity), critical to differences in estimated transmission breakpoint values, implies that the core difficulty in eliminating complex dynamical diseases, such as filariasis, is fundamentally related to the problem of how best to develop elimination strategies in the face of endpoint uncertainty in different sites. While adaptive management strategies, whereby data from each site or from endemically homogeneous regions could be used to develop and apply local strategies, would provide the optimal solution [29,30], this is unlikely to be practically possible in most endemic settings. This implies a need to consider strategies developed and used in other fields (for example, engineering) for managing complex dynamical systems [9,27]. The first of these might be to rely on achieving local elimination on the assumption that good local elimination everywhere implies good ultimate elimination overall, as long as the local interventions and elimination targets are well chosen. This approach could start by splitting the overall problem into a hierarchy of levels, with objectives for local, short-term elimination initially set at a higher level - for example, achieving disease control first [31] - and then expanded on a longer time scale to accomplish parasite transmission interruption [27]. The second tactic may be to avoid focusing solely on meeting the objective of uncertain elimination and exploit the ability of even a relatively poor model to give fairly good guidance to promote good parasite system transitions (for example, parasite control or even elimination) and prevent bad transitions (for example, infection re-emergence following control) [9]. Previously, we have shown that including vector control with MDA can, by increasing the worm breakpoint threshold value, reduce the resilience of the endemic state and, by raising the re-emergence infection threshold, promote the resilience of the parasite-free state, and hence, can play this resilience-enhancing role in sustaining LF elimination [8,32].

## Conclusions

In conclusion, complex parasite transmission dynamics and model or knowledge uncertainty demand the careful consideration of the best management strategy required to achieve parasite elimination both locally and globally. Local dynamics imply different targets for parasite elimination and anticipatory approaches to the management of elimination, based on globally-set thresholds, are unlikely to achieve global filariasis elimination [9,29,30,33]. Urgent work is now required to characterize the nature of variability in local parasite transmission and extinction dynamics using adaptive model-fitting methods, and to test and validate alternative management tactics if we are to develop and successfully deploy a more informed theory of parasite elimination.

## Methods

### Model outline

A population model of infection, using the parasite (*Wuchereria bancrofti) *that causes lymphatic filariasis in the geographic regions for which we have community-level site-specific data, was constructed by extending a set of previously defined coupled partial differential equations [8,22,34]. The state variables of these equations vary over age and time (*a, t*) and represent the adult worm burden per human host (*W*), the microfilarial level in the human host from a 20 μL fingerprick blood sample (*M*), the average number of L3 infective larval stages per mosquito (*L*), and a measure of the experience of infection by human hosts (*I*). The basic model as applied to LF has been discussed previously [8,22,34] and models pertaining to other helminth infections, which have a similar immigration-death structure to the model described here, have also been written about extensively [35,36]. The specific equations of the extended model used here are given in Additional File 1 along with tables giving parameter definitions and value ranges.

Details on the derivation of the effective reproduction number (*R*_*eff*_) and basic reproduction number (*R*_0_) for the model system are also given in Additional File 1. The effective reproduction number, by definition, approaches a value of one at equilibrium and this can be exploited to calculate values for the worm breakpoint. Specifically, the function will intersect the *R*_*eff *_= 1 line twice when a worm breakpoint is present in the system and the value of worm intensity occurring at the lower of these two intersections will be the breakpoint (Additional File 1, Figure 1).

We used the Matlab modelling and analysis package [37] to conduct all the model simulations and analysis described here.

### Populations and data

The data used in this analysis were obtained from endemic communities encompassing the three major geographically distinct regions endemic for LF - PNG, Tanzania and India. Apart from allowing an examination of potential regional differences in the human host response to LF infection [20,24], these data sets were also chosen because they provide a means to examine the theoretical proposition that differences in larval infection dynamics occurring between the two major vector species involved in transmitting LF may also significantly influence elimination thresholds for this parasitic disease [3,7,8], given that the major mosquito vector species involved in parasite transmission in the PNG and Tanzania regions is *Anopheles *whereas *Culex *mosquitoes constitute the major vector in the Indian endemic community. Details of overall ecological, infection and sample size characteristics of each of the study communities are given in Table 1. The data from PNG were obtained from a field study, conducted between 1993 and 1998, of five communities from the Dreikikir area in the East Sepik region of the country for which individual infection and disease status were available for analysis [38,39]; the Tanzanian data were collected from three villages along the northern coastal region and were available aggregated into age-groups [21]; and the data from India were for the population of Pondicherry, southern India, again available for analysis here in age-grouped form [40-42]. In each of these cases, the data components used to fit and test the model were: for the PNG case, the individual binary variable indicating positivity or negativity for infection; and, for the cases of Tanzania and India, the proportion of those within a particular age-group who were found to be positive for infection. The prevalences obtained in the data sets for which a blood sample volume lower than 1 mL was collected were corrected using factors published previously [31].

**Table 1:** Data sets used in this paper. Details of the geographic region, ecology and endemicity of each data set used in the paper.

Region	Village Name	Vector species	Annual biting rate (per person)	% Baseline microfilarial prevalence	No. in sample
Papua New Guinea	Ngahmbule	*Anopheles*	4346	59	285

	Peneng	*Anopheles*	8194	67	63

	Nanaha	*Anopheles*	11611	57	183

	Yauatong	*Anopheles*	37052	92	131

	Albulum	*Anopheles*	42328	80	50

Tanzania	Kingwede	*Culex*	1548	3	825

	Masaika	*Anopheles*	6184	29	848

	Tawalani	*Anopheles*	12850	32	367

India	Pondicherry	*Culex*	69120	14	24677

### Fitting the model to the data and quantifying the uncertainty

The fitting of complex ecological models to data, especially when these models, as in the case of dynamic parasite transmission models, have a relatively large number of parameters with uncertain values, is notoriously difficult [16]. Data fitting by specifying and minimizing an objective function, either defined by a least squares or log-likelihood expression, is problematic because such 'open' models are normally characterized by: (1) the uncertainty about the expected values of key parameters; (2) the existence of many parameter value combinations that minimize the objective function; (3) the minimization of the least squares or negative log-likelihood expression complicated by the existence of multiple local minima; and (4) the fact that the available data may not be detailed enough to support the narrowing of uncertainty in important parameters to a degree that will sufficiently reduce ranges of uncertainty in predicted outputs [13-15].

Here, we used a Monte Carlo-based method developed by Poole *et al*. [43] and recently applied to infectious disease modelling by Spear *et al*. [15,44] - but initially implemented in the context of environmental science [17] - the BM algorithm [43,45] - to address this model-fitting and uncertainty analysis problem. The BM method takes all available prior information on model inputs and outputs and, where available, likelihood functions for data and generates posterior distributions of model inputs and outputs through statistical comparisons of predictions with data. The essence of the method is to initially assign to each parameter of a model a distribution function reflecting the current uncertainty of its value and to refine these estimates from new information, provided by the data. The form implemented here uses (1) uniform or vague prior distributions for each of the model input parameters (except for the ABR, which is fixed to the value measured at baseline in the study data) and (2) likelihood functions for the available data which in the present case are age-prevalences of infection and therefore assumed to be binomially distributed. The multidimensional space defined by the set of prior distributions for each input parameter is then evenly sampled 100,000 times. For each instance of a sampled parameter vector, the model is run and likelihoods are calculated for the age-dependent prevalence curves generated. We then used the SIR algorithm [43] to resample from the original set of 100,000 parameter generates with the probability of acceptance of each resample proportional to its output likelihood value (details in Additional File 1). We resampled to obtain 500 parameter sets that were then used to generate distributions of the desired variables of interest from the model (that is, worm breakpoints, TBRs and *R*_0 _estimates for each of the study communities) and to construct simple post-treatment trajectories. The estimated distributions were then used to quantify the range of uncertainty, given the model, input parameters and data, around each of the above variables for each community.

Comparison of differences between the set prior and model induced posterior parameter distributions of each passing model fit to the age-mf prevalence data from each study community was conducted using univariate Kolmogorov-Smirnov statistics [46], while differences in estimated values of worm breakpoints, TBRs and *R*_0 _values between study sites with different major transmitting vector species were investigated by applying the non-parametric kruskal wallis test.

We used the multivariate classification tree approach to investigate which of the fitted model parameters differed significantly between the study communities and which may, therefore, be considered to underlie any between-community variation in the model outputs [47].

## Abbreviations

ABR: annual biting rate; ALB: albendazole; BM: Bayesian melding; DEC: diethylcarbamazine; LF: lymphatic filariasis; MDA: mass drug administration; mf: microfilaria(e); NTD: neglected tropical disease; PNG: Papua New Guinea; SIR: sampling importance resampling; TBR: threshold biting rate; WHO: World Health Organization.

## Authors' contributions

MG and EM constructed the model and ran the experiments. All the authors wrote and commented on the manuscript.

## Supplementary Material

Additional file 1 **Additional Information**. Model details, parameter values, uncertainty estimation, prior-posterior parameter analysis. Click here for file
