# Supplementary material for: Geographic and ecologic heterogeneity in elimination thresholds for the major vector-borne helminthic disease, lymphatic filariasis
Source: BMC Biol. 2010 Mar 17;8:22. doi: 10.1186/1741-7007-8-22 (PMC2848205; doi:10.1186/1741-7007-8-22)
Supplement: Additional file 1 — Additional Information. Model details, parameter values, uncertainty estimation, prior-posterior parameter analysis. [file 1741-7007-8-22-S1.PDF]

Additional Information

Full model definition

The model may be expressed as a set of partial differential equations in time and age variables:

$$\frac{\partial W}{\partial t}+\frac{\partial W}{\partial a}=ABR\,\psi_1\psi_2 s_2 h(a)L^* g_1(I)g_2(W)-\mu W$$

$$\frac{\partial M}{\partial t}+\frac{\partial M}{\partial a}=\alpha\phi(W,k)W-\gamma M$$

$$\frac{\partial I}{\partial t}+\frac{\partial I}{\partial a}=W-\delta I$$

$$\frac{\partial L}{\partial t}=\lambda\kappa g\int\pi(a)(1-f(M))da-\sigma L-\lambda\psi_1 L$$

$$L^*=\frac{\lambda\kappa g\int\pi(a)(1-f(M))da}{\sigma+\lambda\psi_1}$$

Here, ABR is the annual biting rate per host in the community; ψ_1 is the proportion of infective larvae leaving mosquitoes per bite; ψ_2 is the proportion of these that enter the host; s_2 is the proportion of these that develop into adult worms; $h(a)$ is the age varying exposure to mosquito bites; μ is the death rate of adult worms; α is the production rate of microfilariae per worm; γ is the death rate of microfilariae; δ is the waning rate of the immunity variable; λ is the number of bites made per unit time by a mosquito; κ is the maximum uptake of a mosquito; g is the proportion of mosquitoes which pick up infection when biting an infected host; $\pi(a)$ is the age distribution of humans in the community; σ is the mosquito death rate; and L^* is the infective larval number per mosquito calculated by the final equation. The model also includes a number of density dependences, whose functional forms are detailed in Additional Table 2: $g_1(I)$ is a function describing the host immune response to incoming larvae[1, 2] which acts to attenuate their establishment within the host; $g_2(W)$ describes the suppression of the immune response to parasite establishment[3, 4], which is thought to act when the

community vector infective rate is large; $\phi(W)$ is the worm mating probability; and $f(M)$ is a mosquito function describing the conversion of mf to L3, referred to here as the uptake function.

Details on the derivation of the effective reproduction number (R_{eff}) for the system of equations described above are given below, with the expression arrived at for this variable given by:

$$R_{eff} = \frac{\Lambda \alpha \overline{W\phi(W)} L^{*} \overline{h g_1(I) g_2(W)}}{(\mu + \mu_1)(\gamma + \mu_1)\overline{M}\,\overline{W}}$$

where the constant $\Lambda = \lambda \frac{V}{H} \psi_1 \psi_2 s_2$ in which $\lambda \frac{V}{H}$ represents the observed *ABR*; and μ_1 is the constant death rate of the human host population. The bars over many of the expressions included in the reproduction number denote the average values of these expressions over age in the host population. The effective reproduction number approaches a value of one at equilibrium, by definition, and this feature of the above function can be exploited to calculate values of the worm breakpoint. As noted in the main text, where the function attains a value of 1, the system will be in equilibrium: the upper equilibrium will be the endemic state, and the lower (unstable) point will be the breakpoint (Additional Figure 1).

Model parameter values and density dependent functions

Additional Table 1: Description and values of the parameters of the model

Parameter symbol	Definition	***Typical Values*** *(range of prior distribution [lower,upper])*
**Model parameters**		
λ	Number of bites per mosquito	*[5,10] per month*
V/H	Ratio of number of vectors to hosts	*Adjusted to ensure correct ABR* (λV/H)
ψ_1	Proportion of L3 leaving mosquito per bite	*[0.12, 0.70]*
$\psi_2\ s_2$	Proportion of L3 leaving mosquito that enter host* Proportion of L3 entering host that develop into adult worms (this product is referred to as the 'establishment rate' in the main text)	*[0.00004, 0.004]*
μ	Death rate of adult worms	*[0.008, 0.018] per month*
α	Production rate of mf per worm	*[0.2, 1.5] per month*
γ	Death rate of mf	*[0.08, 0.12] per month*
g	Proportion of mosquitoes which pick up infection when biting an infected host	*[0.26, 0.48]*
σ	Death rate of mosquitoes	*[1.5, 8.5] per month*
δ	Immunity waning rate	*0 per month*
**Model functions**		
k(M)	Aggregation parameter from negative binomial distribution; this consists of a constant, k_0, and a linear component, $k_{lin}M$, dependent upon the mf intensity *M*	$k_0 + k_{lin}$M: *[0.0006, 0.0008]+[0, 0.04]*M
h(a)	Parameter to adjust rate at which individuals of age *a* are bitten: linear rise from 0 at age zero to 1 at H_{lin} years	H_{lin}*: [1, 20] years*

L^*	Equilibrium value of the larval density (see Equation 5)	*Varying*[*]
$\pi(a)$	*Probability that an individual is of age* a	*Varying*[*]

[*] These functions/parameters vary over the course of the simulation or over age

Additional Table 2: Density dependent functions and parameters included in the model

**Density Dependence**	**Expression**	**Parameters**	**Typical Values** (range of prior distribution [lower, upper])
Larval establishment immunity $g_1(I)$ [1, 3]	$\frac{1}{1+cI}$	c - strength of immunity to larval establishment	[0.1, 0.00001] per worm month
Host immunosuppression $g_2(W)$ [3, 5]	$\frac{1+I_C S_C W}{1+S_C W}$	I_C - strength of immunosuppression; S_C - slope of immunosuppression function	I_C: [0.5, 5]<br>S_C: [0.01, 0.19]
Vector uptake $U(M)$ [1]+	$\kappa\left(1-e^{-\frac{rM}{\kappa}}\right)^a$ where $a=1$ for *Culex* and $a=2$ for *Anopheles*	κ - maximum level of L3 given mf; r - gradient of uptake	κ: [3.9, 4.9] (*Culex*) (larvae)<br>[3.6, 4.8] (*Anopheles*)<br>r: [0.17, 0.21] (*Culex*) (larvae/mf)<br>[0.05, 0.06] (*Anopheles*)
Adult worm mating probability $\phi(W,k(M))$ [6]	$1-\left(1+\frac{W}{2k(M)}\right)^{-(1+k(M))}$	$k(M)$ - negative binomial aggregation parameter ($=k_0+k_{lin}M$)	As in Additional Table 1

+ This function differs from *f(M)* included in the larval equation in the main text. The function *f(M)* is obtained when *U(M)*, the conversion of mf density into L3 larvae for a mosquito biting a single individual, is averaged over a population in which the mf are distributed unevenly. When the distribution is negative binomial, we obtain the functions detailed by Gambhir and Michael (Gambhir M, Michael E: **Complex ecological dynamics and eradicability of the vector borne macroparasitic disease, lymphatic filariasis**. *PLoS ONE* 2008, **3**(8):e2874.)

Calculation of the basic and effective reproduction numbers

R_0 is, strictly speaking, equal to zero at the disease-free equilibrium for macroparasitic systems in which there are positive density dependences. We can, however, use the effective reproduction number expression to obtain a measure of the 'raw' reproduction number of these models, unhindered by positive density dependences (dds), following the arguments set out in Regoes et al.[7]. By either assuming a quasi-equilibrium state for fast-changing variables and by finding the largest eigenvalue of the next-generation matrix, we obtain an expression for R_{eff} (appropriately averaged over age):

$$R_{eff} = \frac{\Lambda\alpha\overline{W\phi(W)}L^{*}\overline{hg_1(I)g_2(W)}}{(\mu+\mu_1)(\gamma+\mu_1)\overline{M}\overline{W}}$$

The parameters are as detailed above, with an additional parameter introduced here for the average human death rate μ_1, set to 0.0015 per month (average lifespan of approx 60 years). When we set each of the positive dds to 1 and examine each of the remaining dds at *(W,M,L) = 0* we obtain an expression for R_0.

$$R_0 = \frac{\alpha\lambda}{(\mu_1+\mu)(\mu_1+\delta)}\frac{gr}{\sigma+\lambda\psi}$$

This function was used to calculate breakpoints and TBR values for this paper as follows. Specifically, breakpoints are obtained as those parasite levels at which the function first intersects the $R_{eff} = 1$ horizontal, while TBRs represent those critical ABR values that caused the function R_{eff} to cross the $R_{eff} = 1$ horizontal again at the first point only (see Additional Figure 1).

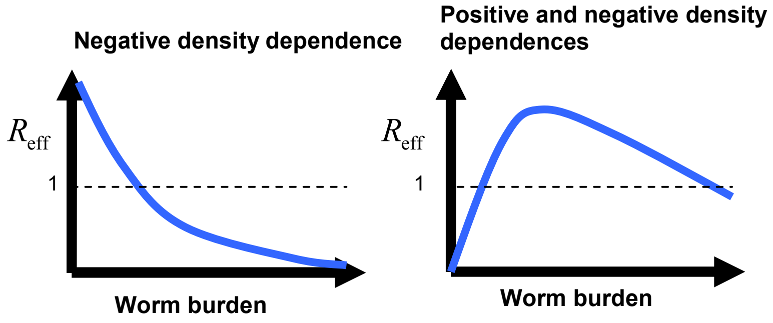

Additional Figure 1 The effective reproduction number. Schematic illustration of the behaviour of the effective reproduction number with increasing mf intensity for the case when a) the density dependences are of a limiting form only, and b) are positive and negative. Equilibria occur when R_{eff}=1, which occurs twice for the facilitation case; the lower equilibrium being unstable and the upper one stable.

Uncertainty estimation

We applied a variation of the Bayesian Melding (BM) algorithm used previously to quantify the uncertainty associated with deterministic model predictions of, for example, oceanic whale population size and HIV prevalence [8]. The original algorithm deals with a deterministic model M that relates a set of input parameters and initial conditions θ, to a set of outputs ϕ, though it has been extended recently to take into account stochastic models[9]. Prior information, based on literature reviews and expert opinion, for both the model inputs and outputs ($p(\theta)$ and $p(\phi)$) are then combined, along with any available data, in the form of likelihood functions for the input and output parameters ($L(\theta)$ and $L(\phi)$). The algorithm we used to quantify uncertainty in the parameters of the present model and hence induced uncertainty in outcomes closely followed the method outlined by Brown et al.[10] :

1) From the prior input parameter distributions, $p(\theta)$, select 100,000 sets of model input parameters.
2) Run the model once for each of the selected parameter sets in order to generate a set of 100,000 model outputs (here microfilarial (mf) prevalence curves).

3) Calculate the goodness of fit for each of the outputs by computing the likelihood for each ($L(\theta)$), given the prevalence data for each endemic area.
4) Resample, with replacement, 500 times from the original set of 100,000 parameter sets, with the probability of drawing each resample proportional to its likelihood for the data calculated in (3).
5) Run the model to calculate the desired quantities for each of the 500 parameter sets found in (4). These quantities are the TBR, breakpoints, R0 values, and, for the full selection of 500 parameter sets, extinction probabilities. As pointed out by Brown et al. [10], it is unlikely that the 500 resampled parameter sets will be unique, since those with the highest likelihood will be picked multiple times in the resampling procedure of (4).

Additional Table 3 Results of the univariate Kolmogorov-Smirnoff (KS) test of differences between the prior and posterior parameter distributions of passing model fits to age-mf prevalence data from each study community.

Village name	Parameter number	Parameter symbol	Median	KS-test p-Value
Peneng	1	β	10.23886	0.059815
	2	α	0.862249	0.73716
	3	k_0	0.000244	0.12207
	4	k_{lin}	**0.010728**	0.036601
	5	k_1	4.419066	0.603374
	6	r_1	0.190052	0.747361
	7	σ_1	5.308408	0.081517
	8	ψ_1	0.40052	0.040564
	9	$\psi_2 s_2$	**0.001178**	0
	10	μ	0.012876	0.431694
	11	γ	0.099062	0.202967
	12	b_1	0.368594	0.585877
	13	c	**0.000804**	0
	14	H_{lin}	**12.61948**	0
	15	V/H	66.69062	0.059815
	16	k_2	**4.335802**	0.000181
	17	r_2	0.132566	0.139618
	19	I_C	**1.292916**	0
	20	S_C	**0.082082**	0.000028
Albulum	1	β	9.805646	0.175748
	2	α	0.619113	0
	3	k_0	**0.000299**	0.757118
	4	k_{lin}	**0.014086**	0
	5	k_1	4.409542	0.686352
	6	r_1	0.1907	0.120279
	7	σ_1	5.014891	0.56961
	8	ψ_1	0.403915	0.086807
	9	$\psi_2 s_2$	**0.001608**	0.009886
	10	μ	**0.013651**	0.002123
	11	γ	**0.103445**	0.000003
	12	b_1	0.37741	0.372124
	13	c	**0.0079**	0
	14	H_{lin}	**12.93503**	0
	15	V/H	359.7261	0.175748
	16	k_2	4.353135	0.171139
	17	r_2	0.133012	0.223401
	19	I_C	**1.811502**	0
	20	S_C	0.095683	0.072914
Yauatong	1	β	**10.17119**	0.000598
	2	α	**0.646652**	0
	3	k_0	0.000503	0.085938
	4	k_{lin}	**0.016922**	0
	5	k_1	4.440266	0.067958
	6	r_1	**0.188243**	0.02397
	7	σ_1	4.797716	0.074348
	8	ψ_1	0.433837	0.158675

	9	$\psi_2 s_2$	**0.002185**	0
	10	μ	**0.014643**	0
	11	γ	**0.104173**	0.000001
	12	b_1	**0.383737**	0.001774
	13	c	**0.008148**	0
	14	H_{lin}	**13.82248**	0
	15	V/H	**303.5697**	0.000598
	16	k_2	4.393252	0.151337
	17	r_2	**0.158881**	0
	19	I_C	**1.002648**	0
	20	S_C	**0.091728**	0.002469
Nanaha	1	β	**9.554677**	0.000015
	2	α	**0.832377**	0.000002
	3	k_0	**0.000296**	0.000001
	4	k_{lin}	**0.00227**	0
	5	k_1	**4.363255**	0
	6	r_1	**0.191383**	0
	7	σ_1	**5.057821**	0
	8	ψ_1	**0.341978**	0
	9	$\psi_2 s_2$	**0.001523**	0
	10	μ	**0.013405**	0
	11	γ	**0.109128**	0
	12	b_1	**0.345178**	0
	13	c	**0.000006**	0
	14	H_{lin}	**2.803097**	0
	15	V/H	**101.268**	0.000015
	16	k_2	**4.428853**	0.000001
	17	r_2	**0.179219**	0
	19	I_C	**0.8119**	0
	20	S_C	**0.133671**	0
Ngahmbule	1	β	**10.50728**	0
	2	α	**0.807199**	0
	3	k_0	**0.000705**	0
	4	k_{lin}	**0.006979**	0
	5	k_1	**4.527248**	0
	6	r_1	**0.187917**	0.0003
	7	σ_1	**4.497186**	0.000434
	8	ψ_1	**0.502186**	0
	9	$\psi_2 s_2$	**0.002028**	0
	10	μ	**0.013673**	0
	11	γ	**0.10299**	0
	12	b_1	**0.37074**	0.000001
	13	c	**0.000005**	0
	14	H_{lin}	**18.00879**	0
	15	V/H	**34.46816**	0
	16	k_2	**4.285744**	0
	17	r_2	**0.155115**	0
	19	I_C	**0.794105**	0
	20	S_C	**0.095831**	0.00091
Kingwede	1	β	**10.30483**	0.00472
	2	α	**1.128977**	0
	3	k_0	0.001004	0.480282

	4	k_{lin}	**0.0029**	0
	5	k_1	4.440532	0.365608
	6	r_1	**0.193748**	0.024161
	7	σ_1	**4.304855**	0
	8	ψ_1	**0.47122**	0.000011
	9	$\psi_2 s_2$	**0.002554**	0
	10	μ	**0.010532**	0
	11	γ	**0.097173**	0.000089
	12	b_1	0.375147	0.058484
	13	c	**0.004671**	0
	14	H_{lin}	**17.00474**	0
	15	V/H	**12.5184**	0.00472
	16	k_2	4.406686	0.683354
	17	r_2	0.139435	0.44648
	19	I_C	**2.75616**	0.012766
	20	S_C	0.093395	0.179401
Tawalani	1	β	**10.42004**	0.000492
	2	α	**1.156932**	0
	3	k_0	**0.001394**	0.004968
	4	k_{lin}	**0.029403**	0
	5	k_1	4.365474	0.385678
	6	r_1	0.187469	0.225904
	7	σ_1	**4.738848**	0.000819
	8	ψ_1	**0.467272**	0
	9	$\psi_2 s_2$	**0.000273**	0
	10	μ	**0.010243**	0
	11	γ	**0.095693**	0
	12	b_1	**0.375908**	0.000287
	13	c	**0.004178**	0
	14	H_{lin}	**17.4565**	0
	15	V/H	**102.7672**	0.000492
	16	k_2	**4.3558**	0.001645
	17	r_2	**0.170567**	0
	19	I_C	**2.17801**	0
	20	S_C	**0.089627**	0.025777
Masaika	1	β	**10.63932**	0
	2	α	**1.135254**	0
	3	k_0	**0.002672**	0.038829
	4	k_{lin}	**0.000647**	0
	5	k_1	**4.486561**	0.046068
	6	r_1	**0.188244**	0.005195
	7	σ_1	**4.641062**	0.000226
	8	ψ_1	**0.452232**	0.000001
	9	$\psi_2 s_2$	**0.002539**	0
	10	μ	**0.011014**	0
	11	γ	**0.095205**	0
	12	b_1	**0.392903**	0
	13	c	**0.000005**	0.034267
	14	H_{lin}	**14.4254**	0
	15	V/H	**48.4367**	0
	16	k_2	**4.320197**	0.000734
	17	r_2	**0.14932**	0.000002

	19	I_C	**1.022451**	0
	20	S_C	0.104514	0.532806
Pondicherry	1	β	**10.95143**	0
	2	α	**0.782494**	0
	3	k_0	**0.000393**	0
	4	k_{lin}	**0.016338**	0
	5	k_1	**4.502677**	0
	6	r_1	**0.198093**	0
	7	σ_1	**4.851834**	0
	8	ψ_1	**0.537531**	0
	9	$\psi_2 s_2$	**0.00019**	0
	10	μ	**0.009273**	0
	11	γ	**0.099532**	0
	12	b_1	**0.370619**	0
	13	c	**0.097105**	0
	14	H_{lin}	**8.843914**	0
	15	V/H	**525.9599**	0
	16	k_2	**4.581539**	0
	17	r_2	**0.081631**	0
	19	I_C	**2.549937**	0
	20	S_C	**0.126419**	0

References

1. Gambhir M, Michael E: **Complex ecological dynamics and eradicability of the vector borne macroparasitic disease, lymphatic filariasis**. *PLoS ONE* 2008, **3**(8):e2874.
2. Norman RA, Chan MS, Srividya A, Pani SP, Ramaiah KD, Vanamail P, Michael E, Das PK, Bundy DA: **EPIFIL: the development of an age-structured model for describing the transmission dynamics and control of lymphatic filariasis**. *Epidemiol Infect* 2000, **124**(3):529-541.
3. Duerr HP, Dietz K, Eichner M: **Determinants of the eradicability of filarial infections: a conceptual approach**. *Trends Parasitol* 2005, **21**(2):88-96.
4. Basanez MG, Collins RC, Porter CH, Little MP, Brandling-Bennett D: **Transmission intensity and the patterns of Onchocerca volvulus infection in human communities**. *Am J Trop Med Hyg* 2002, **67**(6):669-679.
5. Duerr HP, Dietz K, Schulz-Key H, Buttner DW, Eichner M: **Density-dependent parasite establishment suggests infection-associated immunosuppression as an important mechanism for parasite density regulation in onchocerciasis**. *Trans R Soc Trop Med Hyg* 2003, **97**(2):242-250.
6. May RM: **Togetherness among Schistosomes - Effects on Dynamics of Infection**. *Math Biosci* 1977, **35**(3-4):301-343.
7. Regoes RR, Ebert D, Bonhoeffer S: **Dose-dependent infection rates of parasites produce the Allee effect in epidemiology**. *Proc R Soc Lond B Biol Sci* 2002, **269**(1488):271-279.
8. Alkema L, Raftery AE, Brown T: **Bayesian melding for estimating uncertainty in national HIV prevalence estimates**. *Sex Transm Infect* 2008, **84**:I11-I16.
9. Sevcikova H, Raftery AE, Waddell PA: **Assessing uncertainty in urban simulations using Bayesian melding**. *Transport Res B-Meth* 2007, **41**(6):652-669.
10. Brown T, Salomon JA, Alkema L, Raftery AE, Gouws E: **Progress and challenges in modelling country-level HIV/AIDS epidemics: the UNAIDS Estimation and Projection Package 2007**. *Sex Transm Infect* 2008, **84 Suppl 1**:i5-i10.
